# Healthcare-seeking Behaviour among the Tribal People of Bangladesh: Can the Current Health System Really Meet Their Needs?

**DOI:** 10.3329/jhpn.v30i3.12299

**Published:** 2012-09

**Authors:** Syed Azizur Rahman, Tara Kielmann, Barbara McPake, Charles Normand

**Affiliations:** ^1^Centre for Applied Research in Cancer Control, BC Cancer Agency and University of British Columbia, Vancouver BC, V5Z 1L3, Canada; ^2^Institute of International Health and Development, Queen Margaret University, Edinburgh, EH21 6UU, UK; ^3^Health Policy and Management, School of Medicine, University of Dublin, Trinity College, Ireland

**Keywords:** Healthcare-seeking behaviour, Health services, Perceptions, Service delivery, Tribal people, Bangladesh

## Abstract

Despite the wealth of studies on health and healthcare-seeking behaviour among the Bengali population in Bangladesh, relatively few studies have focused specifically on the tribal groups in the country. This study aimed at exploring the context, reasons, and choices in patterns of healthcare-seeking behaviour of the hill tribal population of Bangladesh to present the obstacles and challenges faced in accessing healthcare provision in the tribal areas. Participatory tools and techniques, including focus-group discussions, in-depth interviews, and participant-observations, were used involving 218 men, women, adolescent boys, and girls belonging to nine different tribal communities in six districts. Data were transcribed and analyzed using the narrative analysis approach. The following four main findings emerged from the study, suggesting that the tribal communities may differ from the predominant Bengali population in their health needs and priorities: (a) Traditional healers are still very popular among the tribal population in Bangladesh; (b) Perceptions of the quality and manner of treatment and communication can override costs when it comes to provider-preference; (c) Gender and age play a role in making decisions in households in relation to health matters and treatment-seeking; and (d) Distinct differences exist among the tribal people concerning their knowledge on health, awareness, and treatment-seeking behaviour. The findings challenge the present service-delivery system that has largely been based on the needs and priorities of the plainland population. The present system needs to be reviewed carefully to include a broader approach that takes the sociocultural factors into account, if meaningful improvements are to be made in the health of the tribal people of Bangladesh.

## INTRODUCTION

Bangladesh, one of the most densely-populated areas in the world, has about 160 million people in a land area of 55,598 square miles (147,570 square km) (1). About 1% of the population consists of what are locally termed ‘tribal groups’ due to their distinct and unique languages, cultures, traditions, religions, and customs that are primarily based in Sino-Tibetan ancestry (2). The majority of these tribal groups, or ‘Jummas’ as they are known due to their livelihood by *jhum* (local term for slash and burn cultivation) live primarily in the hilly areas of the southeastern region of the country, specifically Rangamati, Khagrachhari and Bandarban districts of the Chittagong Hill Tracts (CHT) and in the regions of Mymensingh, Sylhet, and Rajshahi (Fig.) (3).

Over the years, displacement and acculturation of the tribal communities have brought about dramatic changes in their lifestyles and value systems. Besides, political disturbances and civil strife during 1974-1996 largely prevented the undertaking of any meaningful development activity in the region (4). The isolation from mainstream development activities, together with a high level of poverty and difficult accessibility to the existing health facilities, made the tribal communities specifically vulnerable to various maternal health problems.

**Fig.**
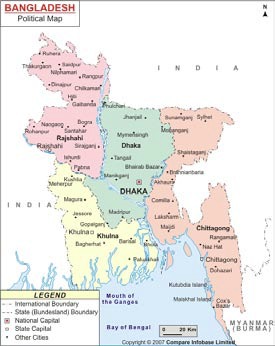
Map of Bangladesh showing the tribal areas

Despite the wealth of studies on health and healthcare-seeking behaviour among the Bengali population in Bangladesh, relatively few studies have focused specifically on the tribal groups in the country. Research on tribal health has predominantly focused on the prevalence of morbidity, profiles of illnesses, and health-provision coverage (5-7) rather than people's knowledge, practices, opinions of and attitudes towards health provision in the tribal areas. An earlier study on health and healthcare-seeking behaviour of the CHT population investigated the prevalence of morbidity and the differences in treatment-seeking among the ethnic minorities and the Bengalis (8). However, it lacks qualitative information on the perceived reasons for their choices of care providers, their knowledge and opinions on health issues and services, and common practices based on the traditional belief systems.

Considering the sociocultural, political, economic and topographical uniqueness of the tribal groups in Bangladesh, their needs of healthcare, attitudes, and healthcare-seeking behaviours may differ from those of the Bengalis (5) and, thus, challenge the present service-delivery system that has largely been based on the needs and priorities formulated by the plainland population.

This paper intends to complement the previous quantitative study (8) as well as provide further insight into sources of treatment, utilization patterns, issues surrounding adolescent and maternal health, and reasons behind preferences for care providers among the selected tribal groups, with the aim of influencing policy formulation, decision-making, and service-provision planning for the tribal population of Bangladesh.

The study aimed to explore the context, reasons, and choices in patterns of healthcare-seeking behaviour of the hill tribal population of Bangladesh. The specific objectives were to: (a) explore the knowledge, practices, and attitudes of the hill tribes concerning their health; (b) examine the availability of service facilities; (c) explore the views of informal care providers (also known as *Baddya*); (d) assess the factors affecting health status of the tribal population and their access to health services; and (e) identify the topographical, cultural and social constraints hindering healthcare-seeking behaviour of the tribal people and their health status.

## MATERIALS AND METHODS

### Study design

This paper is based on a qualitative study conducted in 2004 for exploring healthcare-seeking behaviours of nine ethnic groups living in six districts of Bangladesh. Focus-group discussions (FGDs), in-depth interviews, and participant-observation techniques were used for comparing, triangulating, and filling in information gaps. The field activities were carried out during March-April 2004.

### Study area and population

Six hill districts with tribal communities were chosen for the study. FGDs were held in 16 villages within 15 subdistricts and covered nine different tribal communities (Table 1). The study included 218 participants—72 men, 71 women, 19 adolescent boys, and 56 girls. Villages were selected with the help of various stakeholders, such as local community leaders, administrators, and health professionals. In addition, six remote villages in each upazila were included in the sample.

### Recruitment of participants

The objectives and overall plan of the study were initially discussed with local administrators and government health professionals for suggestions in selecting FGD locations, identifying key informants for interviews, and considering transportation and security issues. The team then met local community leaders to explain the purpose of the study and to identify participants, times, and places for the FGDs. The community leaders spread the message among tribal members and, thus, acted as the main recruiters of participants. As an incentive, participants received Tk 300 (CAD 3.9) and one kg of sugar to compensate for their time. To further support the recruitment process (especially of female participants) and overcome the cultural and linguistic differences between the researchers and the tribal populations, interpreters and a female member from the tribal community were recruited and given basic training. Participants included men and women within their reproductive age-range (13-49 years) mainly to cover the complex physiological and psychological changes that take place during this stage of life (10).

**Table. 1.** Number of FGDs by district, community, location, and type

District	Tribal community	Upazila	FGD locations and group-types with number of participants	
			1. Birishiri—Male: 12
Netrakona	Mandi*	Durgapur	2. Birishiri—Female: 11
			3. Birishiri—Adolescent female: 12
	Chakma	Naniachar	4. Betchari—Female: 12
Rangamati (CHT)	Marma	Bilaichhari	5. Dhigalchari—Male: 13
	Tanchanga	Khawakhali	6. Lungipara—Adolescent female: 13
	Khashia	Panchhari	7. Guchachagram-Female: 12
Khagrachhari (CHT)	Tipra	Dihginala	8. Shanti Laxmipur—Male: 12
	Marma	Matiranga	9. Gagan Chandra Para—Adolescent male: 9
		Lama	10. Masterpara—Male: 12
Bandarban (CHT)	Marma	B. Sadar	11. Ghungru Para—Female: 12
	Khyang		
		Ruangchari	12. Milonpara—Adolescent female: 11
		Ramu	13. Panerchara—Male: 12
		Ukhia	14. Musharkola—Female: 12
Cox's	Bazar	Rakhaine	
	Chakma Teknaf		15. Kharonkhali—Adolescent female 11 Adolescent male: 10
	Tipra Kamalganj		16. Magurchara—Female: 12
Maulvibazar	Khashia		17. Ranirbazar—Male: 11
	Manipuri	Srimangal	18. Dulchara—Adolescent female: 9

The plainland ethnic groups, including Santal, were excluded in this study to keep the sample homogeneous in terms of geographical location;

*The Mandi are commonly known as Garo; however, the people prefer to be called Mandi which means human-being (9); FGD=Focus-group discussion; CHT=Chittagong Hill Tracts

### Focus-group discussions

Four types of guidelines for FGD relating to issues surrounding healthcare-seeking behaviour, attitudes, practices, sources of treatment, and opinions on the quality of care providers were developed. A pilot FGD, with 13 men and women aged 15-49 years, was then conducted in a tribal community in Rangamati district. Based on the findings of the pilot FGD, the guidelines were reviewed, standardized, and adapted.

Four main groups were selected for the discussions. The homogeneity of the participants ensured ease while discussing their knowledge on health, attitudes, beliefs, norms, and common practices and provided an insight into age- and gender-specific attitudes towards health and illness.

A checklist was developed setting out the procedure for documentation of the individual case, and descriptive field-notes were taken to add to the overall picture. Each FGD included a social mapping exercise for participants to understand the topography of the locations of health facilities and their health services and to encourage discussion on accessibility issues. All FGDs were conducted in rural areas where the tribal groups predominantly live. Birishiri in Netrakona district is the only location at the urban periphery.

The research team spent three days, on average, at every location. Initially, the team met community leaders, elites, and influential people in the village and familiarized themselves with the area. The second day was spent on introducing the research team to the potential participants, discussing the objectives and aims of the study, and deciding on the most convenient times and places for the discussions. The FGDs took place on the third day with an average of 12 people participating in each FGD which lasted for 2-3 hours. FGDs were held in easily-accessible locations chosen by the participants themselves and included school buildings, participants’ houses, and tribal cultural centres.

### In-depth interviews

In-depth interviews were held with four participants who had described unusual health-related incidents during the FGDs and seemed keen to provide further information on their experiences. In addition, key-informant interviews were held with five *Baddya* who were asked about their professional qualifications and skills, service-delivery patterns, fees, perceptions of healthcare-seeking behaviour among the tribal communities, and healthcare practices. Informal discussions also took place with formal service providers who were encountered during the fieldwork.

Data sought on the type of healthcare were obtained by asking the respondents about the nature and order of treatment measures undertaken at home or outside their homes. The treatments were grouped into five categories (Table 2).

### Observations

Field observers visited the nearest public-health facilities in the study areas and observed interactions between the patients and the service providers to understand the factors underlying the provider-patient relationship in the facility setting. The observations were conducted in an unobtrusive manner and did not include the scrutiny of care providers from a technical quality point of view, rather concentrated on personal attributes influencing the interactions. The observation field-notes enabled the researchers to compare their impressions with members of the tribal community. Altogether 37 observations were made—six in the upazila health complexes (UHCs) and 31 in the hospitals at the subdistrict level (one in each district).

**Table. 2.** Type of healthcare

Type	Description
Self-care	Instances where no medication was used or where home-remedies were employed
Traditional methods	Treatment-seeking from traditional healers, such as *Baddya,* homeopathic practitioners, and traditional birth attendants (TBAs)
Para-professionals	Village practitioners with basic preventive and curative training on health
Qualified allopaths	Private practitioners, trained physicians working at NGOs, public-health facilities, such as Union Health and Family Welfare Centres, Upazila Health Complexes, district hospitals, community clinics, missionary hospitals, and army physicians
Unqualified allopaths	Drug-sellers, untrained pharmacists, and village *daktars* with little or no professional training, but instead, either self-trained or ‘trained’ by family members

NGOs=Non-governmental organizations

### Formation and training of team

The field research team included three Bengali men and two women who all had a master's degree in social sciences and had experience in field research and qualitative data-collection techniques. Additional training on the specific needs for the study was provided before initiating the fieldwork. Each of them was assigned different tasks in field supervision, moderation of FGDs, interviewing, observations, and taking notes. An additional female team member was recruited from the tribal community. In some cases, several interpreters were hired from the communities to overcome language barriers. The pilot FGD in Rangamati additionally trained and exposed the team members to the practical issues.

### Information management and analysis

At the field level, information from FGDs was recorded through note-taking and audio-recording. Key-informant interviews and in-depth interviews were conducted using the pre-tested interview guides. The field-notes were then compared with the recordings to identify the gaps and errors and also matched with other sources, such as case documents and observation notes to triangulate the information. A logbook was developed for each data-collection technique: FGD, key-informant interview, observation, and in-depth interview. Information was analyzed using narrative techniques to discover “regularities in how people tell stories or give speeches” (11) and find patterns on which we could build our understanding of how participants interpreted their situations. Analysis involved the separation of broad themes relating to the study objectives, subject matters, and concerns of the participants and then synthesizing the information relating to these issues by marking relevant key statements, words, and opinions. A final logbook was prepared outlining the salient information in main themes that had emerged from all three tool-specific logbooks and, thus, provided the main source of information for this paper.

## RESULTS

Views emerged that suggested a prolonged response towards illness and treatment-seeking, leading to delays in the consultation of a health provider of any type. Voicing a general sentiment in response to a question regarding when participants would usually seek treatment, one male participant from the Marma tribe stated: “… treatment was sought two or three days after appearance of signs and symptoms of a disease … especially in the case of fever or stomach problems.” This was confirmed by a healthcare professional at the public hospital in Lama who stated: “They do not come to the hospital until and unless they fall in bed … sometimes, they come to the hospital at the eleventh hour.” However, when an illness was deemed serious enough for treatment, members of the tribal community displayed a strong pattern of preference in their choices of available healthcare providers.

### Healthcare providers and their use

In Bangladesh, like most countries in the South Asian region, a multitude of healthcare providers exists, ranging from specialized modern facilities with physicians trained and qualified in Western medicine to traditional practitioners whose healthcare practices are based on traditional belief systems.

The *Baddya* emerged as the most common source of treatment among the tribal people for most illnesses. A male participant from the Tanchanga tribe said: “… The *Baddya* are our first and last source of treatment.” They are well-represented in the hill villages and seemed to be appreciated for their low costs (payment was often made in the form of gifts or local produces), possibility of delayed payment, and consistent availability. Despite the lack of their medical training, the tribal people demonstrated faith in their spiritual skills passed on from ancestors and spiritual teachers and sought treatment from them for a range of illnesses, such as orthopaedic problems, mental health issues, infections, and common cough and cold. Village *daktars* (a term used in tribal areas to describe local healthcare providers who lack any formal medical training), drug-sellers, and untrained pharmacists were also consulted for the treatment of the common illnesses and, in some cases, they even visited their clients at home when they were too sick to seek care. Homeopathic practitioners emerged as the most commonly-used healthcare providers for women and children and, together with traditional birth attendants (TBAs), seemed to form the most important sources of treatment for reproductive health matters. The tribal villagers relied largely on them for obstetric services and all pregnancy-related issues since the majority of deliveries took place at home. The TBAs were mainly appreciated for their wisdom and skills, easy accessibility, and comparatively low service charges that were supplemented with gifts in the form of wine and handicrafts.

Despite the unpopularity of the military presence in the hill areas due to violent army operations (12), the tribal people considered the services provided by army physicians to be of high quality. In emergencies, patients were sometimes brought to army camps where they were given treatment in tents. The military camps were also approached when there was a need for transport to reach the district hospitals and UHCs. Private practitioners and government physicians who offer their services after office hours at high consultation-fees were equally considered to provide high-quality services. Due to the high costs involved, several male participants of FGDs from Naniarchar, Rangamati, mentioned their reluctance to use them apart from emergency situations. Regardless of their distance and the costs that the Christian missionary hospitals charged for their services, the tribal people who had access to the hospitals appreciated the quality of services, cleanliness, and polite behaviour of the health staff working there. In emergency situations, the respondents communicated their preference for the missionary hospitals or private practitioners over the public-health facilities, particularly Upazila Health and Family Welfare Centres (UHFWCs) and community clinics whose reported irregularities in service provision, erratic availability of physicians, and unofficial fees ultimately led to the decline in attendance by the tribal people.

### Reasons for choices in health provision

#### Financial and logistical obstacles

Costs and accessibility emerged as the important factors from the discussion on sources of treatment. High costs were reported, particularly concerning the purchase of medicines for severe illnesses, e.g. malaria and jaundice (Tk 300-500 for malaria and Tk 800-1,000 for jaundice, which includes the consultation of a *Baddya*) and emergency care at the hospital level. Table 3 provides a breakdown of costs by type of care provider. In addition, transport costs, travel time, and distances formed the important barriers to healthcare-seeking for rural tribal members who had to cover long distances and rugged terrain without regular transport opportunities to reach allopathic healthcare providers usually located in the urban or peri-urban areas of the hill districts. Tribal people of Netrakona, Cox's Bazar, and Maulvibazar were better-off in terms of transport opportunities and road communication. Unqualified providers were mentioned as the most accessible in terms of distance, time input, and familiarity.

Many responses suggested that the participants felt underprivileged compared to the Bengali population in the country. With comparatively low household incomes and dependency on cultivation as the major source of revenue, factors such as fees for care providers, costs of medicines, distances, transportation costs, travel time, and the necessity of accompaniment for women and children, were mentioned as the obstacles to seeking care. As illustrated in Table 3, several sources of treatment in the hill areas, such as private practitioners, missionary hospitals, and even public-health centres, involved cash payments, and participants across all the tribal groups mentioned difficulties in the affordability of modern treatment-seeking. One male participant from the Tipra tribe said: “To treat complicated diseases (surgery cases), we had to sell our valuable properties.” However, views emerged indicating that fees for service providers were not the most significant determinant of choice for healthcare provider. People of the tribal community were willing to pay larger sums for emergency care or even for specialized care from *Baddya* and TBAs, if they felt that the services they were receiving were of high quality, efficient, and respectful of their cultural differences. Accordingly, the missionary hospitals ranked relatively high in their esteem, together with private practitioners despite their higher costs. The district hospital and the UHFWC were ranked lower because of their perceived poorer quality of services, unofficial fees, and shortage of staff and medicines. One male participant from the Khyang tribe said:

**Table. 3.** Fees (in Taka) by type of treatment source and district

District	UHC and DH	UHFWC	Baddya*	Village *daktars*	Private practitioner**	Missionary hospital	TBA	NGO
Rangamati***	Registration fee plus unofficial payment	Officially free but unofficial fees reported	50-300	20-30	100	550 for emergency treatments†	100-300	NA
Khagrachhari***	Same as above	-	50-300	20-30	100	NA	100-300	Tk 20 for initial enrollment and Tk10 for each visit
Bandarban***	Same as above	-	50-300	20-30	100	NA	100-300	NA
Netrakona	Same as above	-	50-200	50	100	100-150, including consultation and medicine†	100-200	NA
Maulvibazar	Same as above	-	50-200	20-50	100	NA	200-300	NA
Cox's Bazar	Same as above	-	100-300	20-30	100	Free leprosy treatment†	200-300	NA

CAD 1=Tk 65 approximately;

*Includes both consultation and medication fees;

**Consultation fee only, which is higher than the fees of *Baddya*;

***Rangamati, Khagrachhari, and Bandarban are within the greater Chittagong Hill Tracts;

†The amount is calculated based on cost of surgery, laboratory, and bed-days. Table indicates costs for most frequent usage;

CAD=Canadian dollar;

DH =District hospital;

NA=Not applicable;

NGO=Non-governmental organization;

TBA=Traditional birth attendant;

UHC=Upazila Health Complex;

UHFWC=Upazila Health and Family Welfare Centre

Doctors are not available at the centre. In the absence of doctors, a laboratory technician provides treatment, which is not good … now, we usually do not visit the UHFWC for treatment.

#### The age factor

Members of the tribal community mentioned the age of the patient and the type and severity of illness as determinants influencing their choice of care provider. Homeopathic practitioners were usually consulted by mothers for the treatment of illnesses of their children. A female participant from the Rakhaine tribe said: “For treating children, we usually go to the homeopathy doctors.” Patients of all ages suffering from diseases perceived as ‘serious’ and as caused by ‘natural’ elements (malaria, jaundice, etc.) were taken to facilities or providers offering modern services. People with illnesses that seemed less serious or were perceived as lacking a direct ‘natural’ cause (headache, bodyache, rashes, etc.) would prefer consulting traditional healers who were appreciated for their extensive knowledge on spiritual elements affecting health and (as the participants stated) whose belief system matched that of their own. Unqualified care providers mentioned their tendency to refer patients to the public-health facilities in cases where they were unable to remedy the illness, indicating an unofficial referral system between the two.

#### Characteristics of care providers

In the discussions and interviews, the tribal people expressed dissatisfaction with the behaviour of physicians at the public-health facilities. The tribal patients felt that the health staff members lacked respect, and they were neither serious nor understood the anxieties of patients regarding their illnesses, unless payments were made. A male participant from the Chakma tribe said: “Only by paying money, you can draw the attention of doctors, nurses, and *Ayahs* of the hospitals.”

A recurrent view provided by FGD participants suggests that the tribal people generally felt that physicians lacked respect and understanding for them due to their cultural differences and mentioned an underlying mistrust resulting from many years of ethnic conflict and tension between the Bengali settlers and tribal communities (9). The differences in ethnicity also caused communication difficulties when tribal patients were unable to speak Bangla.

#### Sociocultural influences

Among the Khyang and Tanchanga communities, numerous rituals influenced the people in their choice of location for the process of healing and treatment. Thus, for example, as explained by members of the Khyang tribe, ill patients who die outside their homes cannot be buried within the family graveyard but are placed along the riverside. The participants of FGDs from the Khyang tribe also mentioned how severely-ill patients belonging to their tribe often choose to stay at home fearing that a change of location could mean the loss of a place within the ancestral graveyards. In addition, responses relating to decision-making power in health-related issues suggest that gender relations influence healthcare-seeking behaviour of the tribes. Among the nine tribes studied, only the Mandi community had a matriarchal system where women have an important role in household decision-making, and men do not have any rights to the family property (9,13,14,15). The patriarchal system predominant in other communities seemed to filter down to decision-making at the family level. Despite the strong presence of women in economic activities, such as agricultural work and selling of commodities, men generally had control over finances and any major decisions made at the household level, including choice and timing of healthcare-seeking. During the FGDs, views suggested that a gender differential exists when prioritizing family members regarding treatment for illnesses. A female participant from the Mandi tribe said: “In case of illness, all members of the family get equal importance … but the husbands and infants/children get more attention during their illness …” Other female participants of FGDs underlined the importance laid on the health of male household heads. A female participant from the Chakma tribe said: “We are very careful about sickness of our husband because if he is confined to bed due to sickness, how do we run the family?”

Overall, gender and age played an important role in determining decision-making power relating to treatment-seeking and perceptions of health. To gain a better understanding of how age and gender influence health behaviour and attitudes, the study paid particular attention to the adolescent, maternal and reproductive health issues.

### Adolescent health

Adolescent boys and girls of the tribal communities showed little interest in their health and how to avoid serious illness. The prevailing attitude among boys was that the likelihood of becoming ill at their age was too little to take seriously. This opinion also surfaced among adults when considering adolescent's health, despite the high prevalence of malaria and other diseases among adolescent boys and girls, especially those living in the hill areas. Young women described problems relating to their menstruation, such as white discharge, excessive bleeding, irregularities in the menstrual cycle, lower abdominal pain, and anaemia. These symptoms were assumed to be part of menstruation and not deemed problematic. As one young female participant from the Chakma tribe described:

Excessive bleeding and severe abdominal pain often happen in my case. At first, I asked my mother about the problem, and she informed me that it is nothing to be afraid of, and it may happen at this age.

Concerning reproductive health, most female participants said that they had the tetanus toxoid (TT) vaccination; however, a few of them could describe the vaccination schedule or seemed to have completed the full course. Despite their motivation to have the TT vaccine, some tribal girls claimed that they could not complete the course of vaccines due to the irregularities of services provided at the satellite clinics in their villages. Knowledge on antenatal and postnatal care was also limited, and most tribal girls mentioned village *daktars*, *Baddya,* and TBAs as the main sources of care in pregnancy. Some adolescent girls voiced concerns about the adequacy of antenatal and postnatal care from the public facilities and mentioned their high cost as a reason for non-use. The adolescents of the Mandi community were better informed about the above issues, which may be due to the cultural activities of the Birishiri Tribal Cultural Centre.

Adolescents generally reported being completely dependent upon the decisions of senior family members for their healthcare and treatment-seeking. The use of modern health services for adolescents was very low, with the *Baddya* being the main source of treatment. Several young women were using homeopaths for menstrual complications. Girls needed to be accompanied by a male relative to the facilities, thus requiring valuable time from a male family member, and it was felt that their families seemed less concerned with their health. Generally, adolescent boys and girls claimed that their families showed little concern with their health and did not prioritize their health issues to spend family resources to pay for treatment.

The overall picture that emerged from conversations with the adolescents suggests that, due to poor access to health information, girls are a vulnerable group in the tribal community that lacks ample opportunities to discuss their health and well-being. It was found that, among the participating adolescent females, the majority (77%, n=56) lacked information on modern healthcare and access to treatments; however, adolescents belonging to the Mandi, Manipuri and Rakhaine tribes had a comparatively better understanding of health and health-related practices.

### Maternal and reproductive health

#### Family planning

Bangladesh has made rapid progress in the family-planning programme. Within the past two decades, the contraceptive prevalence has increased to 53% from 8% (16). However, results of the FGDs and interviews showed a different picture among the tribal groups. The reported use of contraceptives among the tribal communities of the hill areas was relatively low. The results of an FGD revealed that half of the married participants from the CHT did not know about modern family-planning methods, and, in general, women from the remote and rural hill areas were not aware of family-planning messages. In fact, doubts regarding the effects of contraception were voiced, with more than half of the female participants expressing concern over the ‘weakening’ effect of contraceptive methods and others avoiding family-planning methods altogether due to negative associations embedded in religious beliefs and social guidelines. A female participant from the Tanchanga tribe said: “… if I use the pill, I become very weak and cannot work at *jhum* …; so, I stopped taking pills ….”

In contrast, the use of contraception appeared to be comparatively prevalent in the Mandi community, with the majority of the female participants reporting that they use contraception “to keep their family small” for financial reasons. The Mandi women collected contraceptives from Family Welfare Assistants, Family Welfare Centres, UHC, and some purchased pills from medicine shops. The oral pill was the most popular method whereas a few women used copper-T and injection, and one Mandi woman had a ligation. It was also found that the male participants of FGDs were better informed about the methods of contraception, although they were reluctant to use contraceptives, suggesting that this was a concern for women. A male participant from the Chakma tribe said: “If family planning is needed, women will take the contraceptive pill.”

### Antenatal care

It was found that the attitudes of the community people and service providers discouraged the use of antenatal care (ANC) by the tribal women. The sociocultural norms, attitudes, and practices, together with facility-related issues, such as dilapidated buildings and unpredictable service provision, further dissuaded the seeking of ANC. The respondents showed little interest in making use of ANC. Pregnancy was considered a normal event in the life-cycle of women and not deemed to necessitate special care. The experiences shared by the participants of FGDs in Netrakona, Bandarban, and Cox's Bazar underlined the extent to which childbirth is considered a routine event. A female participant from the Tipra tribe said: “… One of my relatives gave birth to her second child while working at *jhumland*.” A female participant from the Tipra tribe stated: “… Just after coming back from the *jhum*, I gave birth at home. No-one assisted me … I did not suffer much.”

Social norms influencing attitudes towards pregnancy discouraged pregnant mothers from changing their daily behaviour due to their condition. In fact, many female participants of FGDs suggested that an improved and more nutritious diet, together with too much resting time for pregnant women, might be hazardous for the mother during delivery of the baby since the foetus may increase in size.

ANC is rarely sought from the public-health facilities; instead, the *Baddya* and TBAs provided the main services. Only those living in close proximity to the UHFWC and UHC reported their occasional visits to the centres for ANC. In the FGDs, members of the Mandi community emerged as the highest users of public-health facilities for ANC. Members of other communities mentioned distance, transportation difficulties, and costs of services as common reasons behind their low use of antenatal services at modern facilities. In addition, lack of knowledge and information on modern ANC and its potential benefits for the delivery outcome, poverty, and gender inequality restricted access to mass media, and the unavailability of services surfaced as important factors.

### Delivery

When prompted on the usual place of their deliveries, women mostly mentioned their home with the assistance of a TBA. The general practice among the tribal groups can be illustrated with this quote from a female participant from the Mandi tribe:

Normally, the deliveries occur at home, and in the case of complications, we usually go to the UHC or UHFWC, private practitioners, or missionary hospitals.

The Marma tribal participants mentioned their beliefs concerning pregnancy-related complications, miscarriage, and stillbirths which were based on spiritual elements, including the ‘existence of an evil spirit’ as stated by a female participant from the Marma Tribe. According to accounts of the participant, once a woman becomes pregnant, she is brought to a *Baddya* who provides her with a *Tabiz* (an amulet containing holy words or a charm against evils) to protect her from pregnancy-related problems. The services of the *Baddya*, appreciated for their special massage techniques, were also sought when women experienced pregnancy-related pains or aches. If complications arose during delivery, family members mentioned their use of incantations of the *Baddya* to protect the mother and the newborn from evil powers. The community members suggested that they were more comfortable in practising delivery-related rituals linked to their belief system in the comfort and familiarity of their own homes.

### Postnatal care

Generally, the tribal people did not use postnatal services unless the mother or the newborn faced any serious problems. Lactating mothers were provided with a better diet and treated with special care. If any problem arose, family members called in a *Baddya*. For serious problems, the patient was brought to the UHC or to the nearest hospital. One FGD participant (a female from the Khyang tribe) shared her experience:

Bleeding started seriously after parturition, and I faced tremendous pain in the whole lower abdomen. Once I became senseless, my husband immediately took me to the UHC, and I was saved.

Specifically among the Khyang community, traditional ways seemed to influence the behavioural patterns after the birth of a child. One FGD participant from the Khyang tribe described a common practice after delivery:

For a newborn son or daughter, the mother would have only fresh rice and salt for six and five days respectively, and during that period, the mother is not allowed to have any fish and meat.

None of the FGD participants mentioned specific reasons for this practice, instead, explaining it in terms of values passed on to them through generations. A female participant from the Khyang tribe said: “… We are just following our ancestors.”

## DISCUSSION

This study explored the treatment-seeking patterns, opinions, and attitudes affecting health and healthcare-seeking behaviour among nine ethnic groups in six districts of Bangladesh. Various medical and quasi-medical options are available for general and maternal and reproductive healthcare. Reasons for pursuing any of these options varied from trust to perception of quality, treatment, availability of resources, cost, and regularity of services. When treatment is sought, traditional medicines and healers still play an important role in maintaining health and well-being among the ethnic groups. Age, gender, and tribal affiliation also affect healthcare-seeking behaviour among the tribes.

### Scope and limitations

We were aware that attitudes of researchers influence the collection and analysis of data; however, we ensured that thoughts and analytical frameworks were shared and discussed among the researchers to minimize bias. The linguistic differences between the tribal communities and some plainland researchers posed initial challenges; however, local interpreters were trained and hired to ease the situation.

The existence and use of a multitude of healthcare providers in Bangladesh has been documented and analyzed in other studies on healthcare-seeking behaviour among the dominant Bengali population (4,17-19). Many of these studies have identified an overall shift from traditional medicines towards modern forms of care (18,20), pointing to the increasingly important role of self-care with modern medicines and a new cadre of ‘para-professionals’ as the main providers of formal allopathic care to the disadvantaged populations (4,17). These shifts in behavioural patterns have been analyzed in the light of microcredit programmes, development interventions, and other health-promoting initiatives that have taken place in Bangladesh over the last few decades and impacted on the accessibility of formal care, health information, and socioeconomic levels (17,18,21,22).

Our findings demonstrate that traditional medicines and healers still play an important role in maintaining the health and well-being of the ethnic groups, particularly in the CHT. While unqualified allopaths were mentioned as a source of treatment due to their easy accessibility and uncomplicated selling of medicines, traditional healers played a much more important role. Among the ethnic groups which participated in this study, the *Baddya* emerged as the most common source of treatment and the first point of referral for any type of illness. This could mainly be due to their low costs and flexibility in payment methods, their strong representation in the tribal villages, consistent availability, and overall accessibility. Accessibility emerged as an important issue for healthcare-seeking in areas that had a poor infrastructure and from practitioners located in semi-urban districts. While costs of service and fees did emerge as a factor underlying treatment-seeking, the perceived quality of treatment surfaced as a more significant determinant. The unavailability of staff, shortage of medicines, unofficial fees, and perceived lack of motivation and respect among health workers were put forward as reasons for the reluctance to use the ‘free’ public-health facilities. Perceptions about the quality of the government health facilities also seemed to be clouded by the underlying tensions between Bengali care providers and ethnic patients who often felt more comfortable with traditional healers embedded with the communities and with the same belief systems.

Previous research also outlines socioeconomic level and education as main determinants in the choice of treatment and care providers accessed (4,18,22-26) as well as severity of illness, accessibility of healthcare provision, and gender (19,21,24,27,28). Thus, higher levels of socioeconomic status, education and severity of illness were positively associated with the choice of formal allopathic care while differences in gender, severity of illness, and accessibility influenced the likelihood of seeking any type of care opposed to self-treatment. Age was not a significant determinant (4), and the factors surrounding the management of healthcare provision and provider attributes, such as their behaviours, characteristics, and methods of treatment, did not emerge as important factors in most studies. Very few studies explored how tribal affiliation could influence healthcare-seeking behaviour.

Our results suggest that gender, age, and tribal affiliation do play an important role in predicting the outcome of choices of households concerning health. Adolescent girls emerged as a vulnerable group without access to health information or advice and were entirely dependent on male members of their households for verdicts concerning their treatment-seeking. Though the findings demonstrate that an overall lack of awareness about health issues, health facilities, their services, and modern treatment methods underlay judgements and health choices among the ethnic groups, interestingly, awareness levels and health knowledge varied between districts and tribal groups.

The Rakhaine, Manipuri, Chakma, and particularly Mandi, seemed to have a different understanding of health and healthy behaviour and were more likely to seek treatment from the local health centres offering modern services. Women belonging to the Mandi community also demonstrated greater awareness about their reproductive health and available methods while the female participants from other ethnic groups underlined their dependency on the decision-making by their male family members for reproductive healthcare. There are several factors that may explain the differences in health-related behaviour and levels of health awareness and knowledge among the ethnic groups. The districts of Netrakona (Mandi), Cox's Bazar (Rakhaine and Chakma), and Maulvibazar (Manipuri) have certain features that contrast with the CHT due to: a more expansive and easier accessible transport system; relative political stability with less ethnic tension between the Bengalis and the tribal groups, leading to more health-promotional activities through NGOs, missionaries, and the Government; and an influx of tourists, thus exposing the tribal groups to different influences and various sociocultural factors, which impact on health beliefs and behaviour.

The use of traditional practices, especially among the Khyang, seems to have an influence on their healthcare-seeking behaviour and treatment of illness. Gender role emerged as an important factor when it came to decision-making concerning health and treatment-seeking. This was felt strongly, especially by adolescent girls from the tribal communities, who felt that they had no say regarding their health and well-being. In this sense, the matriarchal system among the Mandi could account for heightened health awareness and knowledge that this ethnic group displayed in the FGDs among both adults and adolescents. Studies among ethnic groups in India discussed the influences of matrilineal systems on gender roles and educational status (9). It has been argued that women's status and decision-making power in communities are higher in the traditional matrilineal systems (29). Similarly, ethnic groups that follow traditional matrilineal descent, inheritance, and location (the husband moves to his wife's house once married) show the differences in educational levels. As Das suggests, the level of literacy among Khasi women belonging to traditional matrilineal societies is higher than that among women belonging to the ethnic groups undergoing transitions that dissolve the matrilineal system or that did not have one in the first place (30). Some sources that have focused on the Mandi in Bangladesh have noted that the educational levels and literacy rates in this tribe are higher than the national average (13,31). The higher status of girls, their stronger educational level, and their importance within household management, resource allocation, and the social system among the Mandi could offer an explanation for their differing attitude to sustaining health and treatment-seeking.

### Conclusions

This paper provides important insights into potential avenues for policy and education to improve the health of people in tribal areas. It may be beneficial to promote and construct new medical centres that provide government-insured services but these would need to overcome the real and perceived problems of informal charges. Information should be made available, and the tribal people should be encouraged to use it, particularly by new and expecting mothers and sexually-active women of childbearing age. A particular need for increased family-planning methods is clear from the FGD responses. It is also important to note the complexity and various responses from members of different tribal groups, suggesting that a number of approaches should be attempted rather than relying on a single avenue for improvement.

It cannot be stressed enough that, while the type of resources available shape an individual's decision-making, there are many powerful personal and sociocultural factors that play an influential role in this process. If meaningful improvements are to be made in the health of rural people, a broad-spectrum approach is needed that considers all the relevant factors holistically.

These findings clearly show that the present service-delivery system that has largely been placed on needs and priorities formulated by the plainland population needs to be reviewed carefully in order to establish a practical, community-friendly healthcare, culturally-adapted delivery system for the hill tribal population.

## ACKNOWLEDGEMENTS

The study was funded by the Department for International Development (DFID). The authors sincerely thank the tribal people for their valuable time and input and the research team for organizing FGDs in a very hard-to-reach community. The authors also thank concerned personnel of UNICEF, Government of Bangladesh, and colleagues at the Canadian Centre for Applied Research in Cancer Control for their advice and support throughout the study.
